# A Novel Mutation in Maize D1 (Dwarf 1) Confers a Severe Dwarf Phenotype

**DOI:** 10.3390/cimb48060578

**Published:** 2026-06-01

**Authors:** Bingpeng Yang, Yanhui Wang, Yufei Teng, Yan Liu, Xinjun Fan, Qianrui Yang, Na Li, Binwen Shi, Wanchao Zhu, Shutu Xu, Wenqian Mei, Jiquan Xue

**Affiliations:** 1 Key Laboratory of Maize Biology and Genetic Breeding in Arid Area of Northwest Region, College of Agronomy, Northwest A&F University, Yangling 712100, China; bingpeng.yang@syngentagroup.cn (B.Y.); wangyanh@nwafu.edu.cn (Y.W.);; 2 Syngenta Seed Technology (China) Co., Ltd., Yangling 712100, China

**Keywords:** maize, extreme dwarf, *D1*, bulked segregant analysis (BSA) mapping, hormone response

## Abstract

Plant height is a fundamental quantitative phenotypic trait affecting maize (*Zea mays*) planting density and is an important focus in the breeding of varieties suitable for mechanical harvesting. In this study, we found a natural extreme dwarf mutant designated as *d25*, whose dwarf phenotype is controlled by a single recessive gene. The phenotype was restored by spraying with gibberellic acid 3 (GA3), which indicated that the mutant phenotype of the *d25* mutant resulted from mutations in genes involved in the gibberellin (GA) metabolic pathway. Additionally, performing bulked segregant analysis on 30 extreme phenotypic plants in the F2 population (*d25* × P002), we located one major quantitative trait locus (QTL) at chromosome 3 from 9.3 to 11 Mb. In combination with transcriptome sequencing analysis of *d25* and WT plants, we identified the cloned typical plant height-related gene *D1*, whose expression was significantly higher in *d25* mutant plants than that in WT plants. Further analysis revealed that a 275 bp structural variant spanning exonic and intronic regions of the *D1* gene accounted for the dwarf phenotype in the *d25* mutant. Protein prediction revealed that this variant site alters the translated protein sequence of this region, thereby modifying its function and impairing the critical pathway responsible for hormone synthesis, resulting in reduced plant height. These findings indicate that the *d25* mutant may be a novel mutant allele of the *D1* gene affecting maize plant development.

## 1. Introduction

Currently, maize (*Zea mays*) yield constitutes approximately 42% of the world cereal production, and plays a crucial role in global food security, feed supply, and industrial development [1]. Over the past few decades, maize breeding in the United States (US) has been at the forefront of the world. The planting density in the corn belt of the central US has increased at an average rate of approximately 1000 plants per hectare per year, which has contributed to the rapid increase in maize grain yield [2]. To adapt to the increasing demand of the human population and the transformation of crop production models, high-density planting is a promising strategy to enhance crop productivity, especially in maize, the largest yield crop worldwide [3,4]. Therefore, an in-depth analysis of the genetic regulatory mechanisms of maize plant height and the exploration of key gene resources are of great significance for breeding new maize varieties with ideal plant architecture adaptable to high-density planting.

In the last few decades, the introduction of dwarf/semi-dwarf genes into crops during the “Green Revolution” [5] has led to substantial yield increases in rice [6] and wheat [7], such as the *sd*1 gene in rice [8] and the *rht-1* gene in wheat [9,10]. In maize, research studies have made significant progress in understanding its regulatory mechanisms, where the most typical regulation involves auxin synthesis and signaling, primarily gibberellins (GAs), brassinosteroids (BRs), and auxin. A series of classical dwarfism-associated genes have been identified, namely *D1* [11,12], *D2* [13], *D3* [14], *D5* [13], *D8* and *D9* [15], and *D11* [16], which are involved in GA biosynthesis and lead to the dwarf phenotype when mutated. Genes participating in the BR pathways also contribute to plant height regulation, such as *BRI1* [17], *ZmDWF1* [18], and *BZR1* [19]. The *BR2* gene affects plant height through the polar auxin transport [20].

Additionally, studies have shown that various transcription factor (TF) genes are also involved in regulating plant height, including *Dwarf & Irregular Leaf1* (*DIL1*), which encodes an AP2/ERF class TF [21], and *BLH12* and *BLH14*, which encode TFs of the BELL1-like homeobox class, by regulating cell division and elongation to change inflorescence architecture and plant height in rice [22]. The *qPH7* gene/QTL, which encodes an NF-YC TF, regulates cell division and elongation to determine plant height, and the *qph7* mutant in rice shows obvious shortened plant height and shortened internode, while plant height increased in plants overexpressing the *qPH7* gene [23]. Furthermore, genes involved in the CLAVATA (CLV) and WUSCHEL (WUS) pathways have been reported to regulate plant height by regulating the shoot apical meristem, which determines shoot elongation and growth. For instance, the *Compact Plant 2* gene leads to compact growth and reduces stem elongation [24]. Moreover, photoreceptor phyB in maize directly interacts with the transcription factor *LG1* to regulate the protein level of LG1 and the expression level of its downstream gene *HB53* under varying densities and environmental light conditions, thereby achieving precise control over maize plant height and leaf–stem angle formation [25]. Also, there are *TANGLED1* (*Tan1*) and *ZmRPH1*, which encode two microtubule-binding proteins involved in the regulation of the cell cytoskeleton [26]; Z*mEMF1L1*, which encodes a protein homologous to Arabidopsis *EMBRYONIC FLOWER1* (*EMF1*) [27]; and *ZmPYL10*, which encodes an ABA receptor protein [28]. Although numerous plant height-related genes have been cloned, fewer of them have been used in maize breeding due to their pleiotropic role in other aspects of plant development and growth. Thus, it is essential to persistently conduct systematic research on the genetic regulatory network of plant height-related genes and then provide theoretical support and gene resources for molecular design breeding in crops.

In this study, the extreme maize *d25* dwarf mutant plant and its corresponding wild-type (WT) plant were used as the research objects based on the natural mutation *d25* found in a greenhouse. The purpose of this study was to gain a better understanding of the genetic mechanism of maize plant height through phenotypic investigation, hormone response assays, stem cell observation and genetic localization, in order to provide valuable suggestions for future maize breeding studies.

## 2. Materials and Methods

### 2.1. Experiment Materials and Phenotypic Observation

The natural mutant *d25* was initially discovered in the 21CN0025 (WT) maize inbred line genetic background from the company Syngenta in a greenhouse in the Yangling District (Shaanxi province, China) in 2022. Greenhouse conditions were set as follows: a 14 h photoperiod (28 ± 2 °C) and 10 h dark period (18 ± 2 °C). The genetic basis of the *d25* mutant was examined by crossing *d25* mutant plants with the wild-type 21CN0025 (WT), 21CN0308 (P001), and 21CN0311 (P002) in a greenhouse in December 2022. The F1 seeds generated from these crosses were then planted and self-pollinated to produce F2 seeds in a greenhouse from March to September 2023. The plant phenomena of F2 plants in the greenhouse were investigated to verify whether the mutant locus is indeed controlled by a single recessive gene or recessive mutant pattern; statistical analysis was performed using the classic chi-square (*χ*^2^) test in order to provide solid data support and a theoretical basis for further genetic research. In addition, the leaves and roots of seedlings at the two-leaf stage were scanned/imaged using the Wseen LA-S plant image analyzer system (Hangzhou Wseen Detection Technology Co., Ltd., Hangzhou, China).

### 2.2. Gibberellic Response Assay

To evaluate the response of *d25* to gibberellic, we treated these plants with gibberellic acid 3 (GA3) at concentrations of 100, 200, and 300 mg/L, using water as control. GA at each of these concentrations was applied to plants once, twice, or three times at one week interval. Three replicates were performed for each treatment concentration and control, using three individual plants for each replicate to ensure statistical significance. The plant growth status was carefully evaluated by monitoring any visible changes in growth patterns, plant height, or any other phenotypic traits that could be attributed to the GA treatment. Evaluations were recorded on the seventh day after treatment, and images were captured to document the progress of the experiment. All treatments were performed under controlled greenhouse environmental conditions to ensure reproducibility. Through this series of hormone response experiments, we aimed to elucidate GA regulatory mechanisms involved in mutant growth processes, providing valuable insights for subsequent genetic analysis and identification of candidate genes.

### 2.3. Positional Cloning of Maize d25 Mutant

Due to the obvious different plant height at seedling stage (Supplementary Figure S1), we collected fresh leaf samples at the five-leaf stage from plants in the F2 population of the *d25* × P002 cross, and stored them at −80 °C. Among them, thirty plants with height similar to the wild type (WT) were selected from the wild pool, and another thirty plants with height similar to the *d25* mutant were selected from the dwarf mutant pool. DNA from each individual plant in each pool was extracted and mixed at equal concentrations for BSA sequence [29], and then analyzed to identify the molecular basis of the mutant phenotype using the method of mutant mapping by positional cloning (map-based cloning). After extracting genomic DNA from the collected young leaves by a cetyltrimethylammonium bromide (CTAB)-based method, the concentration and quality of the genomic DNA from each sample were determined using a NanoDrop2000 Spectrophotometer (Thermo Fisher Scientific Inc., Waltham, MA, USA). DNA libraries (350 bp) for sequencing on the Illumina (Illumina Inc., San Diego, CA, USA)/Beijing Genomics Institute (BGI) sequencing platform (BGI Group, Shenzhen, China) were constructed for each accession according to the manufacturer’s specifications. After DNA library construction, sequencing was performed on the Illumina HiSeq XTen/NovaSeq/BGI platform by a commercial genomic service provider (Biomarker Technologies (BMKGENE) Co., Ltd., Beijing, China), with 150 bp read lengths.

Raw sequencing data were first subjected to quality filtering using the fastp software (version 1.3.3) to remove low-quality bases and adapter sequences, thus ensuring the reliability of data for subsequent analysis. The clean reads obtained after quality control were aligned to the maize B73 reference genome (AGPv4) using the Burrows–Wheeler Aligner (BWA)-MEM software (version 0.7.19) with the default parameters [30] to determine the physical position of each single-nucleotide polymorphism (SNP) site. The sequence data obtained from the alignment were converted into the appropriate format using the SAMtools software (version 1.23.1) [31], and duplicate sequences generated by polymerase chain reaction (PCR) amplification were removed to avoid interference of duplicate sequences with the results of subsequent SNP variation detection [31]. SNP calling was performed using the Genome Analysis Toolkit (GATK) v4.2.3 software [32]. The following parameters were used for detecting SNP variation sites with the GATK software (version 4.6.2.0): the sequencing depth of the pooled samples was not less than 5× and not more than 100×, and the screened sites were required to meet the polymorphism characteristics among populations. To examine the potential impact of SNP sites on gene function, the analysis of variance (ANOVA) function in the R software (version 4.6.0) was used for functional annotation and effect prediction of the detected SNP variation sites.

In this study, the Δ(SNP-index) indicator, as defined in the mutation mapping (MutMap) method [33], was used to quantify the difference in allele frequency of target SNP sites between the two pools. In addition, further analysis of each SNP variation site was performed using the R software package dQTG-seq to calculate the dQTG.seq1, G’, smoothed logarithm of odds (smoothLOD), ΔSNP-index, and Euclidean distance (ED) values. The window size for the smoothing analysis (Smooth method) was set to 0.5 [34].

### 2.4. RNA Sequencing and Data Processing

Fresh leaf and root tissues were collected from WT and d25 seedlings at the seedling stage, respectively. Each sample had three biological replicates with three plants for each replicate. All samples were immediately frozen in liquid nitrogen and stored at −80 °C for subsequent transcriptome sequencing and qRT-PCR analyses. Total RNA was extracted using prechilled reagents from the Tiangen RNAprep Pure Plant RNA extraction Kit (Cat. No. DP411; Tiangen Biotech Co., Ltd., Beijing, China) following the manufacturer’s protocols. RNA sequencing (RNA-seq) libraries were constructed using an Illumina Standard mRNA-seq library preparation kit (Illumina Inc.) according to the manufacturer’s protocol. RNA-seq libraries were sequenced by paired-end 150 bp sequencing on the Illumina NovaSeq XPLUS platform (Illumina Inc.) using the BMKCloud bioinformatics platform by the genomic service provider, Biomarker Bioinformatics Technology Co., Ltd. (BMKGENE, Beijing, China), and each sample generated 4 Gb of raw data. RNA-seq reads were trimmed using the Trimmomatic (v0.39) software [35] and mapped to the maize B73 reference genome (AGPv4) using the Spliced Transcripts Alignment to a Reference (STAR) aligner software (version 2.7.11b) with default parameters [36]. Unique reads mapped to exons were used for gene expression quantification. The gene expression levels were measured by the fragments per kilobase of exon model per million reads mapped (FPKM) method using the StringTie 2.1.4 transcript assembler software [37]. We used a gene expression threshold of FPKM ≥ 1 as standard to define a gene as “expressed”. The DESeq2 package in the R programming environment was used to identify differentially expressed genes (DEGs, *p* < 0.05 and |log_2_fold_change| ≥ 1) [38].

## 3. Results

### 3.1. Isolation and Characterization of a Maize d25 Mutant

**Figure 1:**
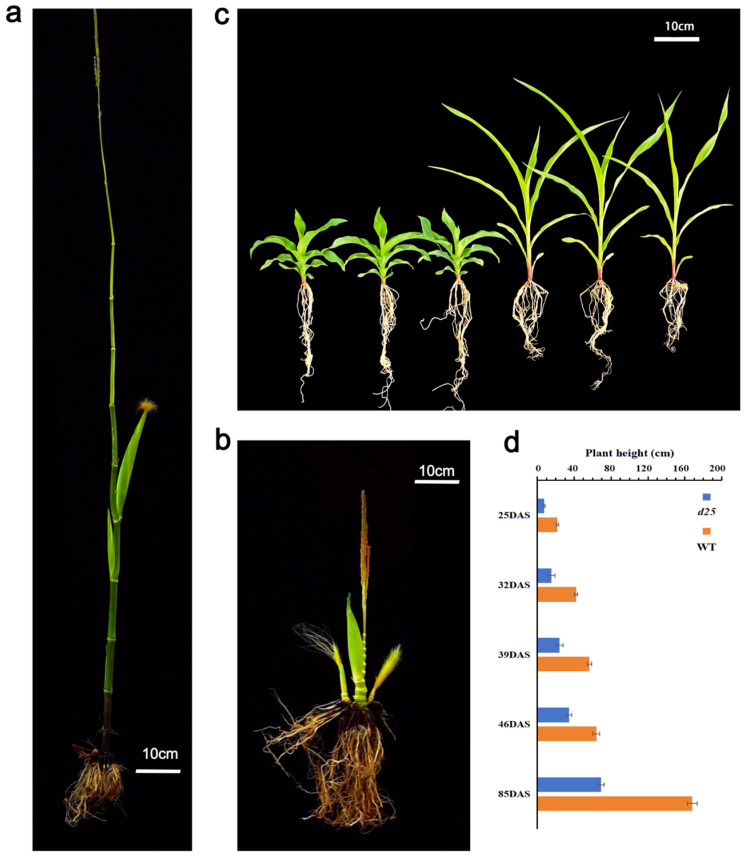
Phenotype of maturing plants and seedlings in *d25* mutant and wild-type (WT) plants: (**a**) WT plants; (**b**) d25 mutant plants; (**c**) WT seedlings and *d25* mutant seedlings at two weeks after sowing; (**d**) the dynamic plant height at different days after sowing.

During the process of seed production of 21CN0025 (WT) in greenhouses, an extremely dwarf maize plant was discovered, which was designated as *d25*. Phenotypic observations of the self-pollinated progeny revealed that *d25* dwarf mutant exhibited a recessive nuclear mutation pattern. Phenotypic identification showed that the height of the *d25* dwarf mutant plant was about 68.92 cm with multiple tillers, while the height of the WT plant was about 167.7 cm without tillers (Figure 1a,b). There was no significant difference in the flowering time between the *d25* mutant and WT plants. The *d25* mutant plant always had two or three ears, which can have normal silking, and the tassel can have more anthers. In addition, we found that the leaf of the *d25* mutant plant had a deep dark green color, while the leaf of the WT plant had a light green color (Figure 1c, Supplementary Figure S1). Additionally, the WT can grow faster and reach a higher height than the mutant plant (Figure 1d).

To determine the genetic basis of the dwarf phenotype caused by the *d25* mutation, we crossed the *d25* dwarf mutant with three inbred lines, namely, its corresponding WT, P001 and P002. The *d25* × WT F2 population consisted of 840 progeny plants, among which 196 had a plant architecture similar to that of the *d25* mutant, while 644 had a plant architecture similar to that of the WT plant. The *d25* × P001 F2 population comprised 978 progeny plants, 218 of which had a plant architecture similar to that of the *d25* mutant, while 760 had a normal plant architecture similar to that of the P001 plant. The *d25* × P002 F2 population consisted of 1000 progeny plants, 225 of which had plant architecture similar to that of the *d25* mutant, while 775 had a plant architecture similar to that of the P002 plant. All of them fit a segregation ratio of 3:1 (*χ*^2^ test, *p* > 0.05, Table 1), indicating that the dwarf phenotype caused by the *d25* mutation is controlled by a single recessive gene.

### 3.2. Response to Gibberellin

**Figure 2:**
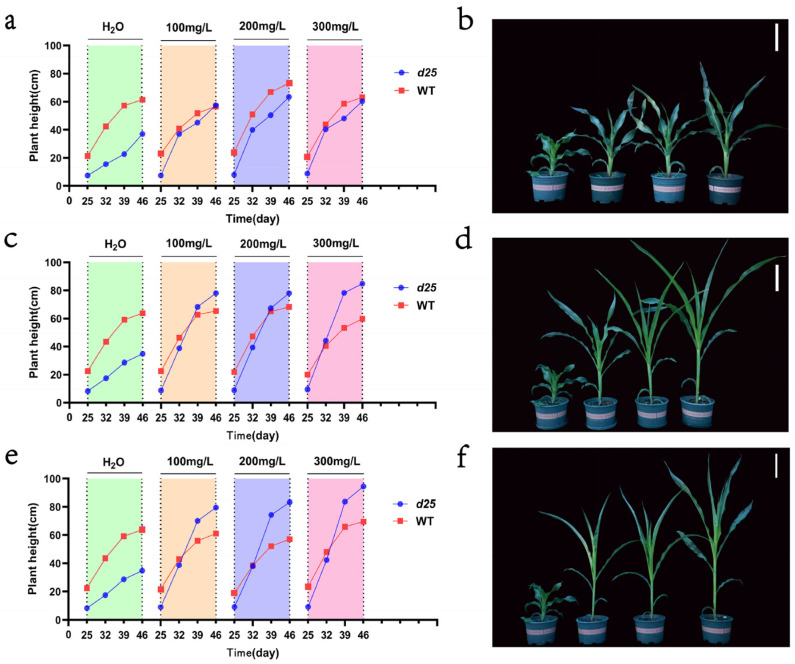
Plant growth performance after treatment with different concentrations of GA: (**a**,**b**) plant growth performance after a single treatment with 100, 200, and 300 mg/L of GA3, or with water as control; (**c**,**d**) plant growth performance after two treatments with 100, 200, and 300 mg/L, or with water as control; (**e**,**f**) plant growth performance after three treatments with 100, 200, and 300 mg/L, or with water as control. Bar = 10 cm.

As most reported dwarf phenotypes result from mutations affecting GA metabolism, specifically those of GA-deficient or GA-insensitive mutants [12], we applied gibberellic acid (GA3) to the *d25* and WT seedlings at different concentrations and frequencies. We averaged the nine plant height values from three replicates in each treatment and performed Student’s *t*-test for two-group comparisons and one-way ANOVA for multi-group comparisons, with *p* < 0.05 considered statistically significant. The results revealed that spraying with GA3 can partially restore the plant height of *d25* mutants to that of WT plants or higher than WT plants, achieving more effective results with batch spraying than with single applications (Figure 2). Longer treatment time led to plants growing taller and thinner. These findings indicate that the *d25* maize mutant responds to exogenous GA3, suggesting that the GA biosynthesis pathway is disrupted in the *d25* maize mutant, but not the GA signal transduction.

### 3.3. Bulked Segregant Analysis (BSA) and Quantitative Trait Loci (QTL) Mapping

**Figure 3:**
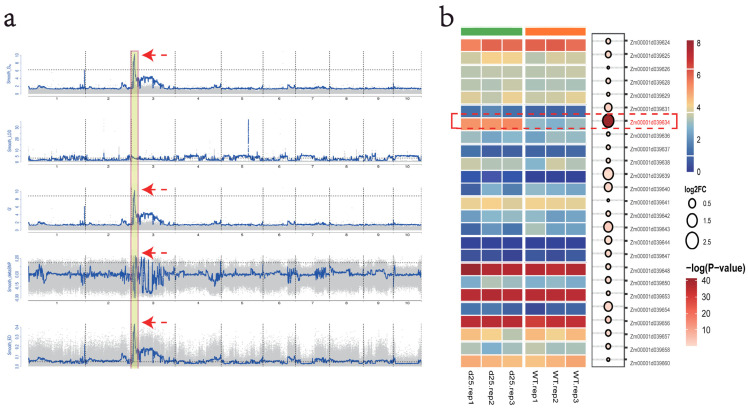
Bulked segregant analysis (BSA) mapping (**a**) and expression profile of the candidate gene located in the QTL region (**b**). The arrow marked the QTL region.

Bulked segregant analysis (BSA) was performed to map the quantitative trait loci (QTL) associated with the dwarf phenotype caused by the *d25* mutant in this study. The experimental materials used were the F_2_ populations derived from the crosses between 21CN0311 and *d25*, namely, 21CN0311 × *d25*, each pool with a sequencing data volume of 72 Gb. The results showed that the mapping rates of the two pools were 99.54 and 99.68%, respectively. In the mutant pool and WT pool, there were 7,226,676 and 7,164,210 SNP loci, along with 1,126,316 and 1,117,588 insertion–deletion (InDel) loci, respectively. Among these variants, 6,462,131 SNP loci and 1,015,113 InDel loci were shared between the two pools. The mapping results using the R software package dQTG-seq identified a candidate region on chromosome 3 ranging from 9.3 to 11 Mb. By integrating the above mapping results, the QTL associated with *d25* was ultimately anchored to the interval of 9.3–11 Mb region on chromosome 3 (Figure 3a). Additionally, the mapping results with MutMap identified one genomic region on chromosome 3 associated with the dwarf trait, with a confidence level exceeding 95%. This region spanned the interval of 10.4–11Mb (Supplementary Figure S2).

### 3.4. Identification of the Candidate Gene

**Figure 4:**
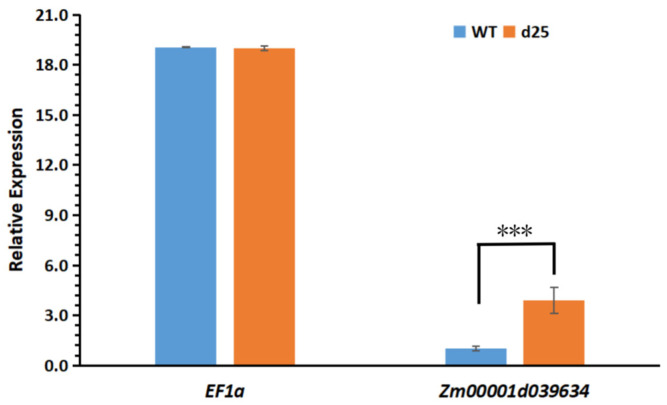
Expression level of Zm00001d039634 (*D1*) determined by qRT-PCR analysis. *** *p* < 0.001.

To identify the candidate gene regulating the maize dwarf mutant *d25*, we performed a comprehensive analysis of the annotations and expression profiles of the 36 genes within the 9.3–11 Mb region on chromosome 3. Comparative transcriptome analysis between the *d25* mutant and wild-type (WT) plants showed that only *Zm00001d039634* (*D1*), a gene encoding a gibberellin (GA) biosynthetic enzyme, displayed significant differential expression, while the other 35 genes showed no differential expression (Figure 3b). However, according to the expression level measured by the FPKM value, the expression level of *D1* in the *d25* mutant was higher than that in the WT plant. This is different from previously reported results, which showed that the dwarf phenotypes caused by *D1* in maize are all attributed to the decreased gene expression levels or premature termination of protein translation [39,40]. Therefore, we examined the gene expression level by quantitative real-time PCR (qRT-PCR) with three biological replicates and four technical replicates, the detail primer sequence was listed in Table S1. Student’s *t*-test results showed that the expression level of *Zm00001d039634* (*D1*) in the *d25* mutant was significantly higher than that in wild-type (WT) plants (Figure 4), which is consistent with the RNA-seq analysis.

### 3.5. Mutation Site in the D1 Gene in the d25 Mutant

**Figure 5:**
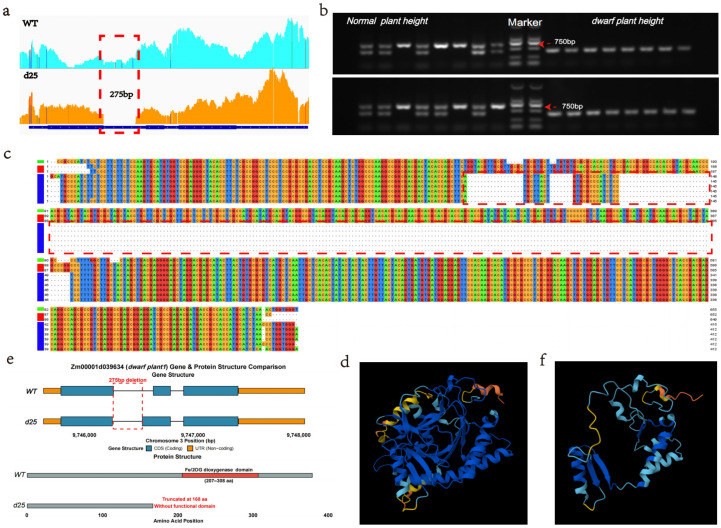
Sequence comparison and marker development for the mutation site: (**a**) comparison of the sequence of the *Zm00001d039634* gene between the wild-type (WT) and mutant (*d25*) plants using bam file; (**b**) polymorphism detected by the marker developed with the WT and *d25*; (**c**) alignment of the sequence of *Zm00001d039634* between the WT and *d25*; (**e**) schematic diagram of the *Zm00001d039634* gene structure and amino acid sequence in WT and *d25*; (**d**) predicted amino acid structure of the *Zm00001d039634* protein in WT; (**f**) predicted amino acid structure of the *Zm00001d039634* protein in *d25*.

Based on the results of the above GA response experiments, QTL mapping, and transcriptome data analysis, we have preliminarily determined that the phenotypic variation of *d25* is influenced by mutations in the *D1* gene. To identify the mutation site responsible for the *d25* phenotype, we aligned and compared the BSA sequencing results using the Integrative Genomics Viewer (IGV) software (version 2.19.7), and detected differences in the *D1* gene between the mutant and WT phenotype pools. The results revealed there was a large InDel between the first exon and the first intron (Figure 5a). Thus, PCR primers were designed for this variant region and the re-sequencing of PCR products showed that there were several InDels (Figure 5b), resulting in a total deletion of 275 bp in the *d25* mutant. Meanwhile, we found that the 275 bp deletion leads to the generation of a premature stop codon. The amino acid sequence of *D1* was predicted using Pfam, and its core functional domain was located at positions 207–308 (Fe^2+^-dependent 2-oxoglutarate dioxygenase). The formation of the premature stop codon may cause the *D1* protein in the *d25* mutant to completely lose its function (Figure 5d). We further predicted the protein structures of *D1* in wild-type (WT) and *d25* plants using AlphaFold3, and a large-scale structural deletion was detected in the *D1* protein of *d25* (Figure 5e). This suggested this 275 bp Indel alters the translated protein sequence of this region, thereby modifying its function and impairing the critical pathway responsible for hormone synthesis, resulting in reduced plant height.

Subsequently, we used this pair of primers to amplify the DNA of F2 populations, a marker that co-segregated with the mutant phenotype (Figure 5c), suggesting that *d1*-506 was responsible for the mutant phenotype.

## 4. Discussion

With the shift in production models from manual to mechanized production, as well as the societal demand for higher yields, increasing maize planting density has become a key issue to be addressed to increase maize production. However, high-density planting imposes strict requirements on maize plant height. Excessive plant height increases the risk of lodging, which can reduce yield, while low plant height reduces field ventilation and light penetration, also limiting yield potential [4]. Therefore, optimizing plant height in maize while ensuring lodging resistance and high yield has emerged as a major scientific problem. In this study, we isolated an extremely dwarf mutant *d25*, and determined, by spraying GA3 treatment, BSA mapping and transcriptome analysis, that a mutation in the *D1* gene is responsible for the mutant dwarf phenotype.

The *D1* is a long-known GA-deficient maize mutant dwarf [13] and has been studied biochemically and physiologically. In the last few decades, Spray et al. [12] reported metabolic evidence that the maize *D1* gene controls the three steps of the GA biosynthesis pathway, namely, GA20 to GA1, GA20 to GA5, and GA5 to GA3, and verified that GA1, GA3, and GA5 were 10 times more bioactive than GA20. Ref. [11] showed that traditional *D1* encodes a GA 3-oxidase for the final step of bioactive GA synthesis and the *D1* protein is dual-localized in the nucleus and cytosol, which suggested that bioactive GA can be synthesized in the cytosol and the nucleus, where the GA receptor is present.

For maize *D1*, various polymorphic variants of this gene were found, which led to a diversity of phenotypes. The first d1 identified was a maize plant with semi-dwarf height [41], but the specific mutation sites were not reported at that time. More recently, Chen et al. [11] found that mutations in *D1* can lead to an extreme dwarf phenotype. In the natural population, QTL mapping for plant height in maize populations generated by using a genetic cross between two natural inbred lines (Zong3 and SL15) identified a QTL, *qPH3.1*, on chromosome 3 within a 12.6 kb interval, which contained *D1* (*ZmGA3ox2*), and found that the natural variant in the promoter was responsible for the different phenotypes in Zong3 and SL15 [39]. Recently, a new semi-dwarf phenotype was identified in D1 and designated as 16N125, which was caused by a single-base mutation (leading to an amino acid substitution at positions 61) in the *D1* gene, and it was found that several inbred lines exhibiting reduced plant height also had alanine at this position, including Si428, Longkang11, Luyuan92, W22, E28, and A188 [40]. In summary, *D1* is an easily mutable gene, and mutations at different sites can lead to varying degrees of dwarfism in plant architecture, as well as other phenotypic variations in maize.

In the *d25* mutant plant, the mutant phenotype is an extremely dwarf height with normal ear and tassel, but multiple branches, which is different from the reported mutant phenotype (semi-dwarf or extremely dwarf height with mutant ear and tassel) in previous studies [11,40,42]. It is also different from the extremely dwarf height mutant designated as *dwarf8* [15], as the *d25* mutant exhibits more severe dwarfism, with broader and thicker leaves (Figure 1b).

To date, four distinct alleles of the *D1* gene have been identified (Table 2): *d1-3286*, *d1-6039*, *d1-4*, and *d1-6016* (Table 2). The *d1-3286* allele contains a 4.5 kb Copia-type retrotransposon insertion at nucleotide position 69 of the ZmGA3ox2 gene [11]. The *d1-6039* allele carries a single-nucleotide deletion at position 399 in the first exon [11]. The *d1-6016* allele harbors a large deletion spanning 2301 base pairs, covering 508 bp of the upstream regulatory sequence and 1793 bp of the *ZmGA3ox2* coding region [11]. The *d1-4* allele contains a 487 bp deletion that affects 389 bp of the first exon and 98 bp of the first intron. All these allelic variations affect the amino acid sequence of *D1* to varying degrees [11]. Among them, the deletion location of *d1-4* is highly similar to that of *d25*, suggesting that the two alleles may reduce plant height through the same molecular mechanism.

Previous studies have reported that *D1* mainly reduces plant height through two mechanisms: decreased gene expression or premature termination of protein translation. Among them, the downregulation of *D1* expression is mediated by *SPL12*, which binds to the promoter of *D1* and represses its transcription, thereby reducing plant height [43]. However, the phenomenon in this study showed higher expression of *D1* in *d25* than in WT. For this, we hypothesize that this 275 bp deletion merely causes premature termination of transcription for this sequence segment, rather than preventing transcription altogether; thus, the sequence remains expressed. However, the protein structure translated from this expressed sequence undergoes alterations (Figure 5d,e), rendering it incapable of participating in hormone synthesis, ultimately leading to impaired hormone production in the plant and reduced plant height. Of course, this conclusion requires further validation through more in-depth molecular genetic experiments.

Due to the complex and cumulative effects of minor polygenes controlling plant height in maize, they are not easily used in production. For instance, the classic plant height gene *BR2* was identified as early as 1935, but the related application system using the *BR2* gene in maize breeding was only established more recently by genetic recombination and editing technology [21,44]. They used genetic recombination, through various hybridization and selection methods, to create a series of dwarf maize inbred lines with the *br2* gene through continuous self-pollination (selfing) and purification [21]. Recently, after selecting gene editing targets using multi-genome alignment and performing selective editing on the fifth exon of *Br2*, researchers found that the gene-editing vector can be used to achieve stalk-lowering improvement in virtually any maize inbred line without decreasing yield [44]. For the *D1* mutant, semi-dwarf and extremely dwarf height have been identified in mutant plants [11,40], and the natural variant in *D1* can also regulate plant height in the natural population [39]. Using a method similar to *Br2*, suitable dwarfing materials with no yield reduction were screened for breeding by constructing saturated editing materials for *D1*. Furthermore, the *ZmGA20ox3* gene knockout (*ZmGA20ox3-KO*) in maize was reported to improve drought tolerance and plant architecture by reducing GA levels and ultimately enhance maize field performance under drought stress, in which the different performances of plant height, flowering time and anthesis–silk interval (ASI) varied dramatically compared to mutant plants under drought conditions [42]. This finding suggested that *ZmGA20ox3* can be used for drought-resistance improvement in maize.

## 5. Conclusions

This study examined the phenotype of a natural extremely dwarf mutant designated as *d25* and its responses to exogenous GA3, and showed that the *d25* mutant is GA-sensitive. Phenotypic characterization in the F2 generation suggested that the phenotype of the *d25* mutant is controlled by a single recessive gene. Bulked segregant analysis (BSA) localized the causal locus to a 1.7 Mb interval on chromosome 3. In combination with transcriptome sequencing of the maize *d25* dwarf mutant and WT plants, only one previously cloned classic plant architecture development gene, namely, *D1* (*Zm00001d039634*), was identified within this interval. A comparative analysis of the *D1* gene sequences between the maize *d25* dwarf mutant and its wild-type (WT) plant identified a 275 bp deletion, which showed co-segregation with the phenotype in the F_2_ population, suggesting this 275 bp deletion might be the causal variant site responsible for the extreme dwarfing phenotype of *d25* in *D1*. There was much work to be done to deepen the understanding of this mutant for uncovering the regulation mechanism. The study enhances the phenotypic variation of the classic dwarf gene *D1* and provides references for further mechanistic study.

## Figures and Tables

**Table 1:** Chi-square (*χ*^2^) test analysis of wild-type (WT) and *d25* dwarf phenotype in three F2 populations.

Population	Normal Plant	Dwarf Plant	Expected Value (Normal:Dwarf)	*p*-Value
*d25* × *WT F2*	644	196	3:1	0.264616
*d25* × P001 *F2*	760	218	3:1	0.050355
*d25* × P002 *F2*	775	225	3:1	0.067889

**Table 2:** All allelic types of the *D1* gene.

Variant	Variant Description	Reference
*16N25*	Alanine replaces proline at position 61; asparagine replaces isoleucine at position 123	[40]
*gad39*	Four InDels and 10 SNPs; nine SNPs in the 5′UTR and coding region lead to four non-conservative amino acid mutations	[42]
*d1-3286*	4.5 kb Copia-type retrotransposon insertion at nucleotide 69 of *ZmGA3ox2*	[11]
*d1-6039*	Single-nucleotide deletion at position 399 in the first exon	[11]
*d1-6016*	2301 bp large deletion covering 508 bp of the upstream regulatory sequence and 1793 bp of the *ZmGA3ox2* coding region	[11]
*d1-4*	487 bp deletion covering 389 bp of the first exon and 98 bp of the first intron; all these variations alter the *D1* amino acid sequence to varying degrees	[11]

## Data Availability

The data presented in this study are available upon request from B.Y. and S.X.

## Supplementary Materials

The following supporting information can be downloaded at: https://www.mdpi.com/article/10.3390/cimb48060578/s1.
