# Supplementary material for: A Novel Mutation in Maize D1 (Dwarf 1) Confers a Severe Dwarf Phenotype
Source: Curr Issues Mol Biol. 2026 Jun 1;48(6):578. doi: 10.3390/cimb48060578 (PMC13298247; doi:10.3390/cimb48060578)
Supplement: Supplementary file 1 [file cimb-48-00578-s001.zip › cimb-4283182-supplementary.pdf]

current issues in
molecular biology

Supplementary figures

Figure S1 Shoots and roots performance at seedlings

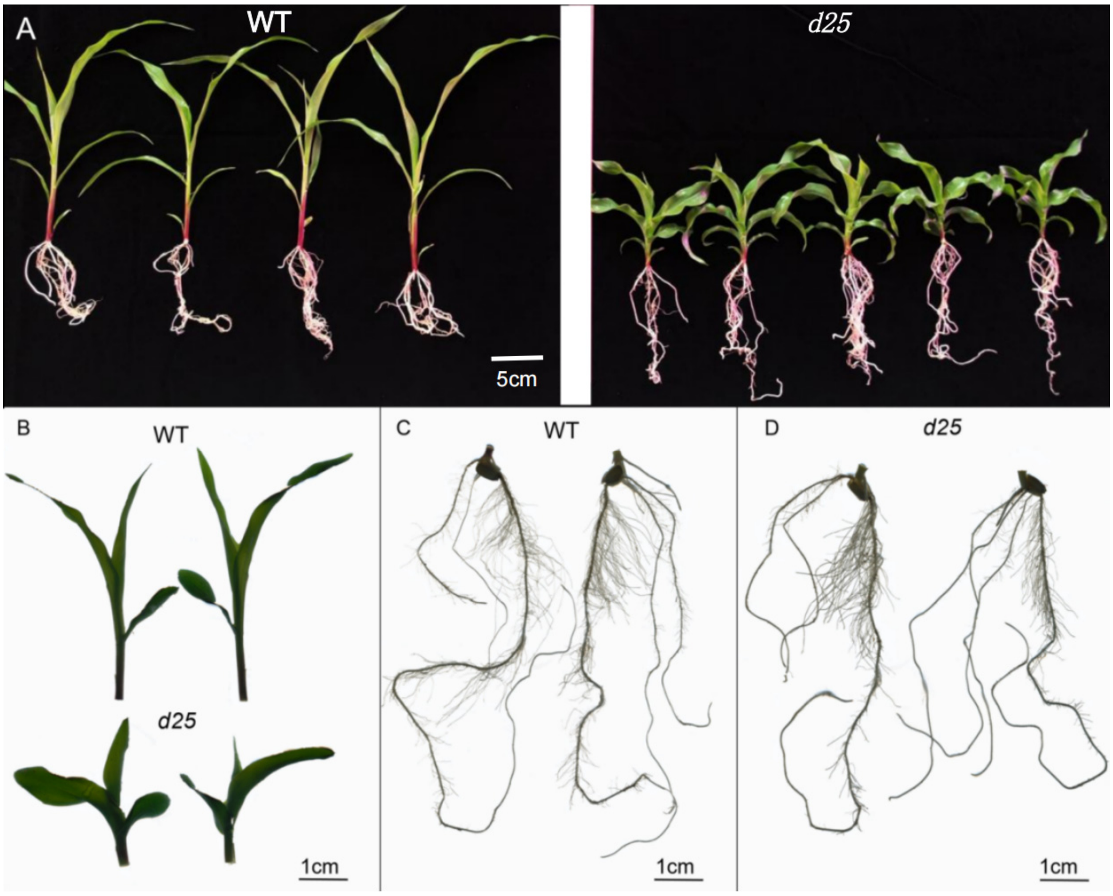

Figure S2 QTL mapping using G value

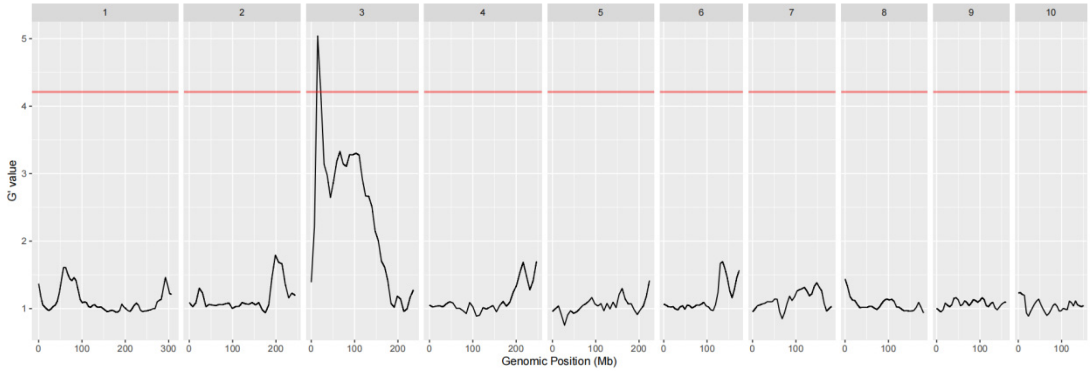

Supplementary Table S1 Primer Sequence for qRT-PCR

Primer name	Sequence 5'-3'	purpose
EF1a-Forward	TGGGCCTACTGGTCTTACTACTGA	qRT-PCR
EF1a-Reverse	ACATACCCACGCTTCAGATCCT	qRT-PCR
Zm00001d039634-Forward	TTCACCTTCGTGATGCAGAG	qRT-PCR
Zm00001d039634-Reverse	GTTGGTGAGGATGTGGAAGAG	qRT-PCR
Zm00001d039634-2F	GCCCATCTCCTCCTTCTTCT	PCR
Zm00001d039634-2R	CCCACCAGTTGAGATGCATG	PCR
